# Micro-anatomic alterations of the placenta in a non-human primate model of gestational protein-restriction

**DOI:** 10.1371/journal.pone.0235840

**Published:** 2020-07-23

**Authors:** James Sargent, Victoria Roberts, Karen D’Souza, Adam Wright, Jessica Gaffney, Antonio Frias

**Affiliations:** 1 The University of Texas Health Science at Houston, Houston, Texas, United States of America; 2 Division of Reproductive and Developmental Sciences, Oregon National Primate Research Center, Beaverton, Oregon, United States of America; 3 Department of Obstetrics and Gynecology, Oregon Health & Science University, Portland, Oregon, United States of America; University of Mississippi Medical Center, UNITED STATES

## Abstract

**Objectives:**

Maternal protein malnutrition is associated with impaired fetal growth, and lifetime consequences for the offspring. Our group has previously developed a model of protein-restriction in the non-human primate, which was associated with fetal growth restriction, stillbirth, decreased placental perfusion, and evidence of fetal hypoxia, suggesting perturbed vascular development. Our objective was to histologically characterize the micro-anatomic alterations associated with adverse pregnancy outcomes taking an approach that permits investigation of the 3D vascular structure and surrounding histology without the requirement for 3D vascular casting or relying on 2D stereology which both have methodological limitations.

**Methods:**

Rhesus macaques were assigned in the pre-gestational period to a control diet that contained 26% protein, or study diet containing 13% protein (50% PR diet). Placental tissue was collected at delivery and processed using a clarification, immunohistochemistry, and confocal microscopy protocol published previously by our group. Three dimensional reconstructions and quantitative assessment of the vascular micro-anatomy was performed using analysis software (Imaris^®^) and statistical analysis accounted for maternal and fetal confounders.

**Results:**

In unadjusted analysis, when comparing those pregnancies on a 50% PR diet (n = 4) with those on a control diet (n = 4), protein-restriction diet was associated with decreased maternal pre-pregnancy weight (difference of -1.975kg, 95% CI -3.267 to -0.6826). When controlling for maternal pre-pregnancy weight, fetal sex, and latency from tissue collection to imaging, a gestational protein-restriction diet was associated with decreases in total vascular length, total vascular surface area, total vascular volume, and vascular density.

**Conclusion:**

In this pilot study, a gestational protein-restriction diet altered the placental micro-vasculature with decreased vascular caliber and density, which may be related to the observed adverse pregnancy outcomes and perturbed placental perfusion previously demonstrated in this model.

## 1. Introduction

Malnutrition continues to be a global issue affecting an estimated 804 million individuals (10.8% of the worldwide population) with areas of Africa, Asia, and South America bearing the brunt of this burden [1]. As early as the 1950s, protein malnutrition was identified as a key factor in global suffering [2], and—although focus on protein-specific malnutrition has waxed and waned over the following decades—recent studies show that protein-specific malnutrition continues to negatively impact human growth and development [3]. In pregnancy, maternal malnutrition has been associated with pre-term delivery, fetal growth restriction, and lifetime cardiac and endocrinologic consequences for the offspring [4–6]. With a high prevalence of protein-malnutrition in the developing world, important knowledge could be gained by understanding its effects on perinatal outcomes.

The placenta is the key mediator between maternal nutritional intake and appropriate fetal development. Placental vascular development is vital to maternal-fetal exchange of gases and nutrients necessary for the development and growth of the fetus; inadequate placentation has been linked to numerous poor outcomes including post-placental hypoxia, fetal growth restriction, pre-eclampsia, and fetal demise [7, 8].

Maternal malnutrition has been found to negatively affect all of the essential placental functions including: establishing blood supply to the developing fetus; gas exchange; nutrient metabolism and transfer; immunologic protection of the fetus from infection and the maternal immune system; and hormone synthesis [9–14]. The extent of protein malnutrition in human cohorts can be difficult to assess due to confounders of diet variation. Use of an experimental animal model can overcome this limitation, with standardized diet composition and monitored intake. Our group has previously developed a model of protein-malnutrition in the non-human primate (NHP) [15] which is highly suitable as a translational animal model due to the similarity in placental vasculature and fetal development [16]. In these well-characterized, highly controlled NHP pregnancies we demonstrated evidence of decreased placental perfusion, fetal hypoxia, fetal growth restriction and stillbirth in those pregnancies exposed to a 50% reduction in dietary protein before and during the pregnancy [15]. Previous efforts to understand the mechanisms underlying how malnutrition can result in poor placental development and function have been limited. The complex interplay between the maternal vascular supply to the placenta and the fetal capillary network, in particular the development of the syncytiotrophoblast as the site of materno-fetal exchange, can be challenging to assess and yet it is of critical importance to our understanding of placental function. Our group has developed a protocol for clarification, which is a method of tissue clearing which renders the tissue transparent for ease of optical imaging, and immunofluorescent labeling of placental tissue [17], which allows for 3-dimensional rendering and the quantitative assessment of the placental vasculature with microvascular resolution, and without the limitations of traditional stereology or vascular casting [18–20].

Using these methods [17], our aim for this study was to histologically characterize the micro-vascular alterations associated with these adverse pregnancy outcomes in our NHP model of gestational protein-restriction (PR). Our prior published work with this model when pregnancies continued to natural delivery at term [15], demonstrated fetal growth restriction by ultrasound biometry at 135 days of gestation, which is the gestational age at which the placental samples were collected in the current study. Having previously demonstrated decreased perfusion with reduced maternal dietary protein content, we hypothesized that by 3D imaging we would find compromised vascular development in the placental villi.

## 2. Materials and methods

### Ethics statement

All experiments utilizing nonhuman primates were performed in compliance with guidelines established by the Animal Welfare Act for laboratory animal housing and care and in accordance with Oregon National Primate Research Center (ONPRC) Institutional Animal Care and Use Committee approved animal protocol (IACUC #1019). The ONPRC is accredited by the Association for Assessment and Accreditation of Laboratory Animal Care (AAALAC) International. Experimental procedures were overseen by veterinary staff, and were designed to minimize potential animal distress, pain and discomfort. Animals were pair-housed to promote social behavior and provided with enrichment devices. All animals were monitored by trained ONPRC clinical, behavioral and husbandry personnel under veterinary supervision, in addition to daily observations by research staff.

### Pregnancy management

The development and findings for our NHP model of gestational PR has been previously published [15]. Placental samples obtained and analyzed for this work were part of a larger animal study examining the impact of gestational protein restriction on maternal, placental and fetal outcomes. All animals had at least one previous pregnancy prior to this study. Female rhesus macaques (*Macaca mulatta*) from the colony at the ONPRC were assigned to either a control chow diet or a 50% protein restricted diet calorie, vitamin, and micro-nutrient matched diet (TestDiet, St Louis, Missouri) at least 3 months prior to timed-mated breeding (TMB) under the management of TMB personnel. Pregnancies were confirmed using early first-trimester 2-dimensional ultrasound (GE Voluson 730 Expert, Kretztechnik, Austria). All pregnancies were terminated by cesarean section delivery at gestational day 135 (where term is 168 days). All surgeries were performed by Surgical Services Unit trained personnel under the supervision of surgical veterinarians in dedicated facilities using aseptic techniques and with animals undergoing continuous comprehensive physiological monitoring. Prior to surgery, food was withheld for 12 hours with no water restriction. Animals were initially sedated with 10mg/kg ketamine given by intramuscular injection for intubation with an endotracheal tube for sedation maintenance under inhaled (1–2%) isoflurane anesthesia. In preparation for surgery, the abdomen was shaved and a local blocking agent (0.8ml bupivacaine (0.5%) combined with 0.2ml lidocaine (1%) with epinephrine) was given intradermally at the incision site. Following intravenous catheter placement, animals received 0.025–0.2mg/kg hydromorphone. Animals were placed in dorsal recumbency and the abdomen was cleaned with a chlorohexidine/alcohol instant solution prior to sterile draping. Standard hysterotomy was performed to deliver the fetus and allow collection of placental tissue. Fetuses were immediately euthanized by trained personnel in the ONPRC Pathology Unit according to the recommendations of the American Veterinary Medical Association 2013 Panel on Euthanasia. Post-operative analgesia was provided to all dams for a minimum of 48 hours with 0.05–0.4mg/kg hydromorphone HCl administered intramuscularly three times daily and 0.01–0.1mg/kg buprenorphine given once intramuscularly for overnight analgesia. Post-operative monitoring and assessment of pain and distress was provided by surgical veterinary staff for a minimum of seven days. Observations of species-specific behaviors such as food and water intake, and urine and feces production were performed daily. Dams were returned to the ONPRC colony following completion of the study. Placental villous tissue was extensively sampled from multiple cotyledons to provide good representation across the whole organ. Biopsy depth was not accounted for but a consistent sampling regime was followed and villous tissue was further dissected into 1mm^3^ sections and placed in 10% ZnFormalin for 24–48 hours prior to storage in 70% ethanol at 4°C.

### Tissue clarification and immunofluorescent labeling

Our tissue clarification and immunofluorescent staining protocol has been previously described elsewhere [17]. In brief, placental villous tissue was clarified using an ethanol-based solvent (Visikol^®^ HISTO2^™^) which renders the tissue transparent to aid immunofluorescent staining and visualization of structures. Fetal endothelium was stained with a monoclonal mouse-anti-human CD31 antibody (Thermofisher, MA1-26196), and the trophoblast was stained with a polyclonal rabbit-anti-human CK7 antibody (Abcam, ab103687) for confocal imaging.

### Tissue imaging

Clarified and immunolabeled samples were placed in a 96-well glass bottom dish with 0.17 +/-0.005mm cover glass (Cellvis, Mountain View, CA), and immersed in 200 μL of Visikol^®^ HISTO2^™^ solution. Imaging was performed using a Spinning Disk Confocal Microscope (Zeiss/Yokogawa CSU-X1, Thornwood, NY) with a 20x Plan-Apochromat, N.A. 0.8 air objective, with two-channels (Leica/ALEXA 488 at 100% intensity and exposure time 250ms, Leica/ALEXA 647 at 50% intensity and exposure time 150ms) in a standardized field size of 488μm x 325μm x 694μm, and optical sections at optimized intervals calculated by the imaging system (0.54μm per slice). Negative controls (no primary antibody, no secondary antibody, or omission of both primary and secondary antibodies) were used to assess for non-specific fluorescence and background auto-fluorescence. Image files were acquired with Zen 2.6 software and saved as a single .czi file prior to being split into individual files.

### Data acquisition

Due to the novelty and complexity of the data acquisition and quantitative analysis protocols development, a thorough outline of these methods is provided in the Supplement of this manuscript (S1 File). Therein variables of interest are defined and an explanation of their quantification method is detailed.

In brief, files were uploaded to Imaris^®^ 9.2.1 software (Bitplane AG, Zurich, Switzerland) and converted to .ims format. All data acquisition occurred on a custom-built Dell Precision Tower 7910 workstation with an Intel Xeon CPU E5-2623 v4 2.60GHz dual processor with 192GB RAM. Once uploaded into Imaris^®^, z-stacks were cropped to isolate the area of interest. Pre-processing steps were minimized to prevent loss or alteration of data: a linear stretch was performed to utilize the entire voxel intensity histogram (0 to 65,535), and a background subtraction with a large filter width. Sample quality was assessed for intensity of fluorescence; where autofluorescence was high and vasculature could not be adequately distinguished, samples were excluded from analysis (n = 3).

Using the filament module, a pipeline was created to minimize processing parameters between tissues. A single starting point was selected subjectively for each tissue, and a seed point minimum of 5.00μm was set based upon the size of the smallest structure of interest (i.e. capillary diameter). Within the filamentation module of Imaris^®^, the “no loop” algorithm was used, and the variables of interest (total vascular length, total vascular surface area, total vascular volume, total number of termini, median branching angle, sholl Intersections) were collected and exported for analysis. A full description of the measured and calculated variables of interest are included in the Supplement (S1 File). Maternal and pregnancy data was compiled including: maternal age at delivery, maternal pre-pregnancy weight, maternal gestational weight gain, maternal weight at delivery, fetal sex, fetal weight, placenta weight, fetal:placental weight ratio, gestational age at delivery, and latency from tissue collection to imaging.

### Statistical analysis

Statistical analysis was completed using GraphPad Prism version 8.2.0. Descriptive statistics (compared using student’s t-test, with significance threshold of p < 0.01), histograms, correlations (compared using Pearson’s correlation with a threshold p<0.01), and univariate linear regression methods were utilized to assess relationships between variables of interest. A multiple linear regression model was generated to isolate the effect of group assignment on when controlling for multiple covariates chosen based on either a detected significance with the univariate analysis or based on previous studies.

## 3. Results

A total of 11 pregnancies (6 in the Control group and 5 in the 50% PR group) progressed to scheduled cesarean delivery. Following completion of the tissue clarification, immunofluorescent labeling, imaging, and filamentation process, data from 4 pregnancies in each group (1 image stack per placenta) was included for the final analysis. Those pregnancies that yielded imaging results of insufficient quality to distinguish between autofluorescence and immunolabeled structures which precluded the generation of reliable quantitative data, were excluded.

**Fig 1:**
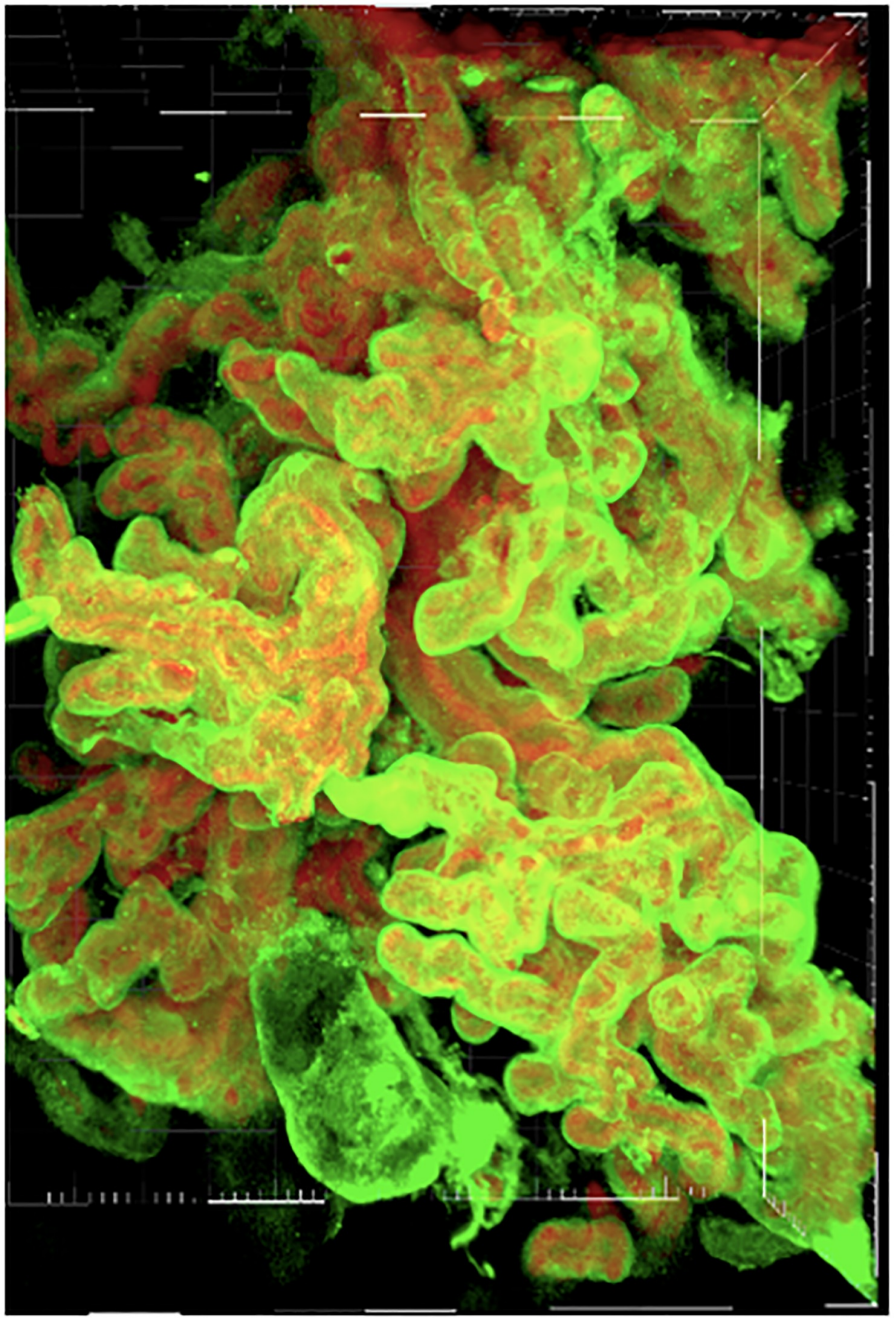
Individual histograms depicting the cumulative percentage of sholl intersections with increasing distance from the origin. PR50—50% protein-restriction.

Maternal, pregnancy, and fetal outcome data was compiled (Table 1, Fig 1), and group analysis using a Student’s t-test revealed a significant difference in the latency from tissue collection to imaging (524 days for the 50% PR group Vs 651 days for the Control, p = 0.0007). Maternal pre-pregnancy weight was close to statistical significance between the two cohorts (5.78kg for 50% PR dams Vs 7.80kg for Control dams, p = 0.018).

**Table 1:** Animal cohort data.

Demographic Data									
Group & Animal ID	Control				50% Protein-Restriction				p-value
	C1	C2	C3	C4	PR1	PR2	PR3	PR4	
Maternal Age (y)	7.8	8.7	7.8	7.8	8.3	8.4	7.3	7.1	0.41
Pre-pregnancy Weight (kg)	8.9	6.6	7.9	7.7	6.1	5.5	5.3	6.3	0.018
Gestational Weight Gain (kg)	2.3	0.2	1.8	1.3	2.1	2.0	1.1	0.7	0.88
Maternal Weight at Delivery (kg)	11.1	6.8	9.7	9.0	8.2	7.4	6.4	7.0	0.12
Fetal Sex	F	F	F	M	F	F	M	F	1.00
Fetal Weight (g)	389.0	298.0	341.6	343.2	312.7	362.0	309.4	257.7	0.41
Placenta Weight (g)	118.7	78.8	106.7	105.0	110.9	74.5	89.6	80.8	0.060
Fetal:Placenta Weight Ratio	3.28	3.78	3.2	3.27	2.82	4.86	3.45	3.19	0.59
Gestational Age at Delivery (d)	138	137	135	132	136	132	135	137	0.83
Days From Tissue Collection to Imaging (d)	652	648	631	674	529	515	526	528	0.0007*

Table: Demographic data for individual study animals. C—Control; PR—Protein-Restriction.

*—significant to p<0.01

**Fig 2:**
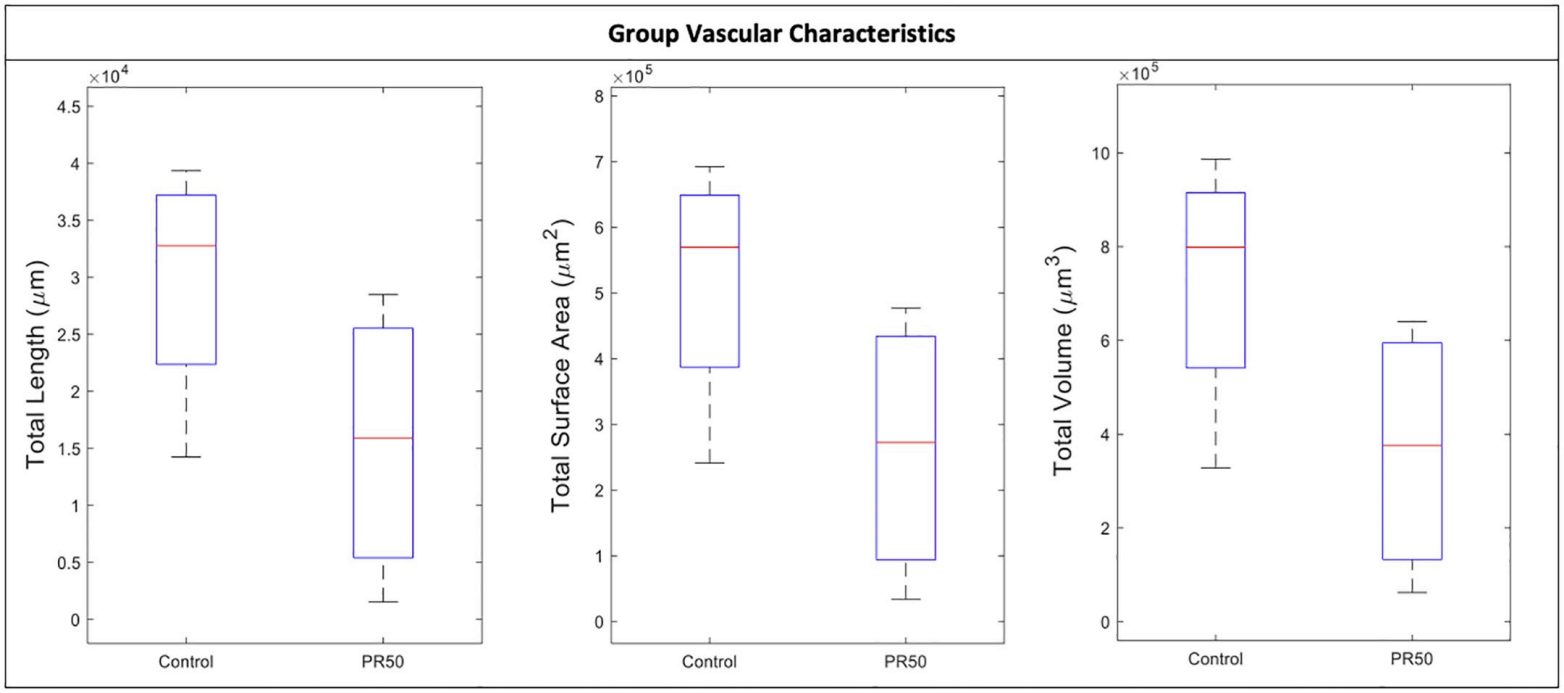
Comparison of group vascular characteristics. PR50—50% protein-restriction.

In group comparison using a student’s t-test (Table 2, Fig 2), none of the vascular variables were significantly different between groups. The rate of increase of vascular density (i.e. sholl intersection—rate of change maximum) was 0.009 intersections per μm for the 50% PR group Vs 0.006 intersections per μm for the Control, p = 0.31.

**Table 2:** Group comparison of vascular data.

Vascular Data									
Group & Animal ID	Control				50% Protein-Restriction				p-value
	C1	C2	C3	C4	PR1	PR2	PR3	PR4	
Total Length (μm)	30468	35127	14277	39353	28484	1555	9250	22600	0.14
Total Surface Area (μm^2^)	533227	605288	241046	692390	476647	33991	153119	391639	0.12
Total Volume (μm^3^)	754529	843215	327973	986492	640395	62199	203311	549369	0.10
Number of Termini	1002	1176	480	1152	1005	63	330	701	0.18
Median Branching Angle (°)	47.4	48.0	35.9	32.0	40.5	13.5	28.1	38.8	0.31
Sholl Intersection									
Cumulative Sum	18452	21710	9803	26256	18168	1052	6647	14249	0.15
Max Sholl Distance (μm)	354	346	476	504	359	151	249	400	0.09
Rate of Change—Maximum (Number / μm)	0.007	0.007	0.006	0.004	0.006	0.017	0.009	0.004	0.31
Rate of Change—Median (Number / μm)	0.0014	0.0015	0.0009	0.0014	0.001	0.0046	0.0012	0.002	0.32

Table: Vascular data for individual study animals. C—Control; PR—Protein-Restriction

In the unadjusted univariate analysis (Table 3), a 50% PR diet was associated with lower maternal pre-pregnancy weight (decrease in weight of 1.975 kg, 95% C.I. 0.68 kg to 3.27 kg), and significantly less latency from tissue collection to imaging (decrease in latency of 126.8 days, 95% C.I. 103.7 to 149.8 days). Correlation tables for the variables of interest displayed the most significant correlations between the variables total vascular length, total vascular surface area, total vascular volume, number of termini, and sholl intersections—cumulative sum.

**Table 3:** Univariate analysis.

Results for Univariate Analysis			
Outcome	β	Standard Error	95% Confidence Interval
Total Length (μm)	-14334	8234	-34482 to 5813
Total Surface Area (μm^2^)	-254138	141914	-601390 to 93113
Total Volume (μm^3^)	-3.64E+05	1.98E+05	-847707 to 119240
Number of Termini	-428	263	-1071 to 215
Median Branching Angle (°)	-10.6	7.4	-28.8 to 7.6
Sholl Intersections—Cumulative Sum	-9026	5172	-21681 to 3628
Maximum Sholl Distance (μm)	-130	69	-300 to 40
Sholl Intersections—Maximum Rate of Change	0.003	0.0029	-0.0042 to 0.010
Sholl Intersections—Median Rate of Change	0.0009	0.00084	-0.0012 to 0.0030
Maternal Age (y)	-0.25	0.4	-1.2 to 0.74
Maternal Pre-Pregnancy Weight (kg)	-2	0.53	-3.27 to -0.68
Maternal Gestational Weight Gain (kg)	0.075	0.56	-1.30 to 1.46
Maternal Delivery Weight (kg)	-1.9	0.97	-4.28 to 0.48
Fetal Sex (Male)	-1.30E-20	0.35	-0.87 to 0.87
Placental Weight (g)	-13.35	11.57	-41.65 to 14.95
Fetal:Placental Weight Ratio	0.2	0.47	-0.94 to 1.34
Gestational Age at Delivery (d)	-0.5	1.7	-4.68 to 3.68
Latency from Tissue Collection to Imaging (d)	-126.8	9.4	-149.8 to -103.7

Table: Unadjusted univariate linear regression exploring the relationship between a 50% protein-restricted diet and outcomes of interest.

In the multivariate analysis (Table 4), after controlling for the impact of maternal pre-pregnancy weight, fetal sex, and latency from tissue collection to imaging, a 50% PR diet was associated with a significant decrease in total vascular length (a decrease of 1.13 x 10^5^ μm in the 50% PR group, 95% C.I. 1.150 x 10^4^ μm to 2.16 x 10^5^ μm), total vascular surface area (a decrease of 1.99 x 10^6^ μm^2^ in the 50% PR group, 95% C.I. 3.96 x 10^5^ μm^2^ to 3.58 x 10^6^ μm^2^), total vascular area (a decrease of 2.81 x 10^6^ μm^3^ in the 50% PR group, 95% C.I. 8.79 x 10^5^ μm^3^ to 4.74 x 10^6^ μm^3^), and sholl intersections—cumulative sum (a decrease of 69,987 intersections in the 50% PR group, 95% C.I. 4,106 to 135,868). All four of these outcomes were significantly correlated with each other (p<0.01).

**Table 4:** Multivariate analyses.

Results for Multivariate Analysis			
Outcome	β	Standard Error	95% Confidence Interval
Total Length (μm)	113714	32131	11459 to 215969
Total Surface Area (μm^2^)	1987983	500389	395522 to 3580444
Total Volume (μm^3^)	2.81E+06	6.06E+05	879281 to 4738205
Number of Termini	3465	1269	-574 to 7503
Median Branching Angle (°)	43.4	61.1	-151.2 to 238.0
Sholl Intersections—Cumulative Sum	69987	20701	4106 to 135868
Sholl Intersections—Maximum Rate of Change	-0.019	0.025	-0.097 to 0.059
Sholl Intersections—Median Rate of Change	8.03E-05	0.0078	-0.025 to 0.025

Table: Multivariate linear regression exploring the relationship between a 50% protein-restricted diet and outcomes of interest. The model was corrected for Maternal Pre-Pregnancy Weight, Fetal Sex and Latency from Tissue Collection to Imaging.

## 4. Discussion

In our NHP model of gestational PR, we have demonstrated a significant decrease in fetal vascular caliber (total length, total surface area, total volume) as well as the cumulative vascular density within the placenta. In univariate analysis, there were significant associations between animal group and maternal pre-pregnancy weight as well as the latency from tissue collection to imaging. These outcomes were therefore included in the multivariate analysis, as well as fetal sex, which is a known confounder, in order to control for their influence.

These findings are consistent with, not proof of causation of, the adverse pregnancy outcomes noted in the parent study [15] where adverse placental perfusion was demonstrated. While we were able to detect significant associations between maternal diet and the placental micro-vasculature, our findings have many strengths and limitations that must be understood.

Like many studies involving the NHP, we are limited by small sample sizes. The benefit of using NHP pregnancies results in high-quality data from highly-controlled, well-characterized pregnancies in an animal model that is similar to that of the human. However these benefits come at the expense of statistical power and potentially can lead to a failure to detect a real difference between groups (Type 2 error). That a difference was detected in this pilot study despite the small sample size is suggestive of a possible association, however this would need to be confirmed in a larger study.

For the purposes of this study, we designed a novel protocol and the strengths and limitations of these methods are outlined in detail in the Supplement (S1 File). Importantly, further validation of these methods will come with time, refinement and utilization.

With the development of our tissue clarification and immunofluorescent labeling protocol [17] as well as our data acquisition and quantitative analysis protocol, there was considerable latency (500 to 600 days) from the time of tissue collection to imaging. Due to how the pregnancies were randomized, this latency was not equal between the two animal groups and we used a multivariate regression analysis to attempt to control for any effect of prolonged storage prior to immunolabeling. The impact of latency on measured structural outcomes for this method is not known. In the future, application of these methods to fresh tissue, or processing the tissue a standard time post-collection, will be important in controlling for this confounder.

Following the imaging protocol, we were unable to accurately quantify the volume of space occupied by the tissue (a full discussion of the technical issues is included in the Supplemental S1 File). This prevented us from being able to assess many of the density calculations that could be of interest (e.g. number of branches/μm^3^, vascular surface area/μm^3^, etc.), however, Imaris^®^ is able to provide a measure of the branching density from the origin with a measurement of “sholl Intersections”. While this measurement does not provide any information about vessel caliber, we were able to detect a significant decrease in the cumulative number of sholl intersections between the groups, and the molecular mechanism underlying this difference could help to further explain the adverse pregnancy outcomes noted in animals fed a 50% PR diet.

The Imaris^®^ filamentation process has its own limitations. Recognizing that this module was developed for the assessment of neuronal axons, the fundamental assumptions of the algorithm have to be understood and accounted for when applying this to vasculature.

The “Loops” algorithm within the filamentation module is not compatible with large datasets, and it’s reliance upon absolute-threshold differences is unsuitable, without additional correction filters, for deep tissue imaging and the inevitable decrease in stain intensity that worsens with increasing depth of imaging that occurs secondary to light-scattering. Secondary to these limitations, the “No Loops” algorithm had to be utilized and this results in some inaccuracies: a single vascular loop at the villous terminus would be modeled as two separate vascular branches which approach each other to create an incomplete loop. This artifact would be anticipated to affect all imaged tissues equally but would decrease the accuracy of any “per segment” analysis as well as any assessment using the number of termini.

Additionally, the Imaris^®^ filamentation algorithm is unable to account for higher order branching patterns. Instead, Imaris^®^ will create a binary branch followed by another binary branch, and this introduces a small vascular segment between the branches. By artificially incorporating an additional vascular segment, the branching depth variable is offset and therefore made less reliable. This artifact affects all tissues, and could bias the datasets collected if test conditions resulted in the tissue having unequal propensities for higher-order branching patterns.

Lastly, due to the errors within the Imaris^®^ vascular segmentation, branching depths, and tissue density data, we were limited in our ability to normalize the data between biopsies to account for z-stacks containing different amounts of tissue. While no significant difference was detected in the unadjusted size of the vascular trees assessed, those variables that were different between the groups (e.g. total vascular length, total vascular surface area, total vascular volume, and sholl intersections—cumulative sum) are all vulnerable to inaccuracy simply due to differences in the amount of a vascular tree captured in a z-stack as opposed to true micro-anatomic variance secondary to maternal nutrition. Further refinement of the quantitative analytic methods will need to address these issues in future studies.

## 5. Future directions

Through the publication and dissemination of our work we hope to encourage other groups to utilize and refine the protocols included and advance the field of quantified analysis. The importance of this method is that it allows for the 3D reconstruction of the villi, which cannot be achieved with 2D stereology. It also preserves the surrounding cellular structures, which is not typically possible with most vascular casting methods. Further validation will come through the comparison with established stereology and casting techniques, as well as through the application to different animal models of placental dysfunction. As more is learned about the micro-vasculature, additional antigens can be stained allowing for the assessment of molecular mechanisms driving observed changes. Lastly, we suggest that future application of this 3D imaging methodology to placenta biopsy samples obtained through chorionic villous sampling during ongoing pregnancies will greatly improve our ability to detect at-risk pregnancies which may facilitate clinical management and improve pregnancy outcome.

## Supporting information

S1 File Data acquisition and quantitative methods. This supplement provides a detailed description of the methodology and analysis parameters used within the Imaris^®^ software to generate quantitative data from our placental samples.
(DOCX)

S1 Fig (TIFF)

S2 Fig (TIFF)

S3 Fig (TIFF)

S4 Fig (TIFF)

S5 Fig (TIFF)

S6 Fig (TIFF)

S7 Fig (TIFF)
