# Correction: Micro-anatomic alterations of the placenta in a non-human primate model of gestational protein-restriction

**DOI:** 10.1371/journal.pone.0242769

**Published:** 2020-11-17

**Authors:** James Sargent, Victoria Roberts, Karen D’Souza, Adam Wright, Jessica Gaffney, Antonio Frias

Fig 1 is incorrect. Please see the correct Fig 1 here.

**Fig 1:**
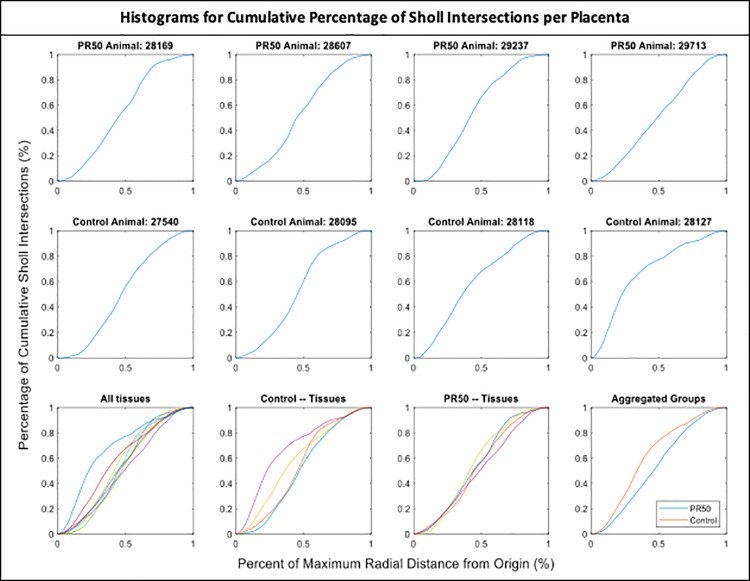
Individual histograms depicting the cumulative percentage of sholl intersections with increasing distance from the origin. PR50–50% protein-restriction.
