# Supplementary material for: Micro-anatomic alterations of the placenta in a non-human primate model of gestational protein-restriction
Source: PLoS One. 2020 Jul 23;15(7):e0235840. doi: 10.1371/journal.pone.0235840 (PMC7377450; doi:10.1371/journal.pone.0235840)
Supplement: S1 File — This supplement provides a detailed description of the methodology and analysis parameters used within the Imaris® software to generate quantitative data from our placental samples. (DOCX) [file pone.0235840.s001.docx]

**Supplemental: Data Acquisition and Quantitative Methods**

1. **Objective**

Using our previously published protocol [1], we are able to immunofluorescently label the vasculature and surrounding villous tissue of clarified non-human primate (NHP) placental tissue. These image stacks are of sufficient quality to acquire vascular parameters (e.g. vessel length, vessel surface area, vessel area, branching angles, vascular density, etc.), and subsequently allowing for quantitative comparative analysis.

The objective for this protocol was to develop a reliable and reproducible technique for the 3D rendering and quantitative assessment of the placental micro-vasculature using Imaris®. Our hypothesis was that we would be able to detect a between-group difference in the rate of increasing vascular density (as measured by the change in cumulative number of sholl intersections moving from the origin of the vascular tree to the distal termini).

1. **3-Dimensional Rendering and Data Acquisition**

2.1 – Tissue Preparation and Imaging

Tissue was collected and immunofluorescently labeled as previously described (Supplemental A) prior to being imaged in a 96-well glass bottom dish with 0.17 +/-0.005mm cover glass (Cellvis, Mountain View, CA). Imaging was performed using a Spinning Disk Confocal Microscope (Zeiss/Yokogawa CSU-X1, Thornwood, NY) with a 20x Plan-Apochromat, N.A. 0.8 air objective, with two-channels (Leica/ALEXA 488 at 100% intensity and exposure time 250ms, Leica/ALEXA 647 at 50% intensity and exposure time 150ms) in a standardized field size of 488µm x 325µm x 694µm, and optical sections at optimized intervals calculated by the imaging system (0.54µm per slice). All tissues were imaged as a single batch-Image file with Zen 2.6 software and saved as a single .czi file prior to being split into individual files.

2.2 – Data Acquisition

Numerous computer programs have been created to process confocal images and allow for image optimization, 3D rendering, and subsequent analysis. Imaris^®^ is a proprietary, stand-alone, image-rendering and quantification software that has been used for over a decade for validated image visualization and analysis. Numerous filters allow users to enhance the signal-to-noise ratio of their images, and to provide reproducible and quantified data on distances, surface areas, volumes, and branching patterns (Table 1). Imaris^®^ is able to provide statistics for each vascular segment (i.e. the segment of tissue that originates at a branching point and then terminates with either a branching point or a free end) allowing for precise characterization of the vascular micro-anatomy. From these, additional variables can be calculated (Table 2) to normalize and aggregate the large amounts of data provided.

Table 1: Variables of Interest


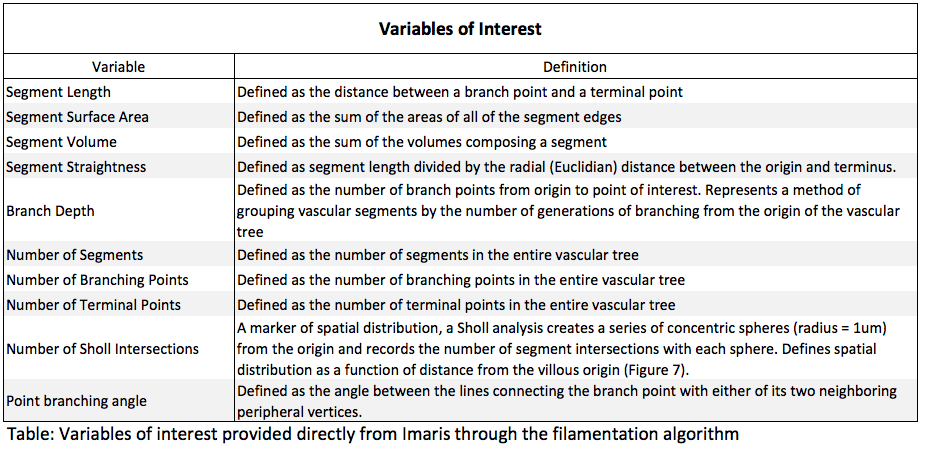


2.2.1 – File Preparation and Pre-Processing

Files were uploaded to Imaris® 9.2.1 software (Bitplane AG, Zurich, Switzerland) and converted to .ims format. All data acquisition occurred on a custom-built Dell Precision Tower 7910 workstation with an Intel Xeon CPU E5-2623 v4 2.60GHz dual processor with 192GB RAM. Once uploaded into Imaris®, z-stacks were cropped to isolate the area of interest (Figure 1).

Prior to quantitative analysis, the signal-to-noise ratio of the z-stack had to be optimized in order to increase the reliability of the subsequent data collection. At the same time, in order to minimize data alteration or loss, the fewest steps with the most limited settings were utilized. For this pipeline, each z-stack was cropped, underwent a linear stretch, and then a background subtraction.

Figure 1

Figure 1: Maximum-intensity projection of immuno-fluorescent labeled (CK7 - green; CD31 - red) and clarified NHP placenta imaged at 20x magnification and imaged using Imaris v9.2.1.

Due to the large size of the files, the z-stack was first cropped to the smallest rectangular field that would accommodate the entire block of tissue. When cropping the visual field, the tissue of interest is held in a fixed position; depending upon the orientation, equal-sized tissue pieces may be in cropped z-stacks of different sizes containing varying amounts of empty space.

As a part of the filamentation process, intensity thresholds help guide vascular identification and diameter calculation. However, during imaging, each collected z-stack has a similar but not identical range of voxel intensity values. In order to create a pre-processing pipeline that could be used for all of the tissue biopsies, the intensity range of each z-stack had to be standardized without changing the relative intensity differences within each image. Therefore, a linear stretch was performed to utilize the entire voxel intensity histogram as set by Imaris® (0 to 65,535).

Background subtraction is a blurring or noise-decreasing step, which applies a Gaussian filter to the entire z-stack and determines a background intensity to be subtracted from each voxel. Gaussian filters smooth image contours, but data loss can be minimized by using a larger filter width which is calculated by the software (rolling ball >100µm for most tissues), and – for this protocol – the recommended rolling ball size was used.

2.2.2 – Filament Generation

As one of its functionalities, Imaris® has a “Filaments” module that was designed to identify and analyze neuronal bodies and axons in imaged tissue. The filaments module can be used to analyze vasculature, but the two available algorithms make assumptions that must be taken into account when applying these tools on non-neuronal structures.

As it automatically identifies and traces the vasculature, the “no loops” local intensity contrast algorithm produces a tree-like filament with dendritic branches and distal spines. This is appropriate for vascular trees given the assumption of a larger starting point that extends, subsequently branches and arborizes into smaller terminal points. Alternatively, the filament module has a “loops” absolute intensity threshold algorithm which is more appropriate for the analysis of small capillary beds, which the “no loops” algorithm cannot capture, however its reliance on an absolute intensity threshold is less well-suited for deep tissue imaging, where the inevitable scattering of light causes progressive decreased voxel intensity from tissue further from the laser source. Importantly, the “loops” algorithm is currently incapable of the parallel processing required for large-scale tissue sizes making this unsuitable for the aims of this study.

While tissue biopsy size was standardized through the use of a core biopsy tool, not all z-stacks contained the same volume of tissue. Additionally, the microscopic anatomy included was variable between biopsies (i.e. not all biopsies contained the same number of villous networks, and not all villous trees extended from stem villus to termini). To account for this, the decision was made to identify a single villous tree per z-stack for analysis; thereby allowing us to explore the relationships between vessel segment measurements at various depths, and the ways those measurements change moving distally from the villous origin to the termini.

Important to the aims of this analysis, Imaris® has the ability to save the parameters used for filament generation, allowing for reproducible results with minimal subjective input from the user. The only subjective element of our data collection pathway was the selection of the single villous tree for analysis; Imaris® is unable to gauge the quality of staining and the size of candidate villous trees to the same degree as the human eye.

Lastly, due to the large, complex vascular networks of interest for this study, certain features of the filaments module (e.g. dendritic spine detection or manual filament tracing) were neither applicable nor feasible.

2.2.2.1 – Step 1 of 6 – Algorithm Selection

Following the pre-processing steps (Figure 2), the filament module was initiated for automatic creation of a filament using the “no loops” algorithm, and the option to “calculate Diameter of Filaments from Image” was selected. For filament generation, Imaris® must first identify – using size and intensity parameters – where each filament begins (i.e. the origin), the path it follows (with each vascular segment defined as the path connecting branching points), and the diameter at each point on the filament.

Figure 2: Maximum-intensity projection of NHP placenta with the vascular (CD31-red) channel isolated, the visual field cropped, and following a background subtraction (RB=112um). In the lower-right field the vascular starting point (blue sphere) was selected manually to guide the filamentation process.

2.2.2.2 – Step 2 of 6 – Dendrite Points Diameter

Within Imaris®, each vessel diameter can be explored by the user prior to beginning the automatic filament generation algorithm. Based on these values, the dendritic starting point (i.e. the origin of vascular tree; the largest diameter) and seed point (i.e. the smallest vessel diameter) sizes were determined, and – for standardization – were set at 15 um and 5 um respectively.

2.2.2.3 – Step 3 of 6 – Classify Dendrite Points

The Imaris® suggested seed points were removed, and a single starting point was selected manually by subjectively assessing each vascular tree for the brightest and most consistent staining throughout the length of the entire network.

When setting the seed point intensity threshold, those voxels below the threshold would be interpreted as background noise and not be incorporated into proposed vascular pathways. To maximize signal intensity at this point in the algorithm, a low intensity threshold of 4500 (~5% of the maximum voxel intensity) was selected (Figure 3).

Figure 3: Maximum-intensity projection of the vasculature of the NHP placenta (CD31-red). Absolute threshold seed points have been selected with an intensity treshold of 4500 to guide the identification of vascular pathways extending from the selected starting point (blue sphere).

For neuronal analysis, Imaris® provides the option of removing seed points that are below a certain distance from each starting point as a way of preventing the algorithm from being confused by bright staining that is within the neuronal cell body. This option was not selected for this analysis.

As a means to increase the specificity of the proposed vascular pathways, there is an option to “remove disconnected segments” which eliminates misdirected branches that inappropriately tried to connect discontinuous vascular segments. As a part of this process, Imaris® performs a temporary smoothing step based on the size of the smallest structure expected in the z-stack (i.e. 5um capillary diameter), and can perform a transient background subtraction to refine the removal process. In order to avoid the inappropriate incorporation of discontinuous vascular segments, the decision was made to include the “remove disconnected segments” option with the transient background subtraction.

2.2.2.4 – Step 4 of 6 – Remove Disconnected Segments

For the “remove disconnected segments” pathway, a background subtraction intensity of 500 was selected in conjunction with a maximum allowable gap distance of 20.0 um. Both of these settings were set permissively in the understanding that – at the conclusion of the automatic filament generation pathway – inappropriately incorporated vascular segments could be removed manually as needed.

2.2.2.5 – Step 5 of 6 – Calculate Dendrite Diameter

Once the pathway from starting point to termini was determined by Imaris® (Figure 4), the vascular diameter at each voxel was then calculated based on a local intensity contrast value of 5 and using the “shortest distance from distance map” – as opposed to the “approximate circle of cross section area” – algorithm. In confocal microscopy, one of the visual artifacts that can be minimized but not removed is the point spread that results in an inappropriate elongation of the visualized object in the z-plane. When Imaris® calculates the vascular diameters, the “shortest distance from distance map” algorithm is not influenced by any point spread which persists.

Figure 4: Maximum-intensity projection of the vasculature of the NHP placenta (CD31-red) with the preliminary vascular skeleton (grey) overlying the native stain.

2.2.2.6 – Step 6 of 6 – Identify Spines

The spines feature is not applicable to the analysis of vasculature, and was therefore omitted.

2.2.2.7 – Smoothing Contour & Final Verification

Following generation of the vascular tree, the filament tool kit contains a smoothing feature which can minimize the changes in vessel diameter within each vascular segment which can occur as a result of non-uniform staining. The “Process Intensity-Based” center option was selected with a minimum expected diameter of 5um (Figure 5).

Figure 5: Maximum-intensity projection of the vasculature of the NHP placenta (CD31 - red) with the modeled vasculature (grey) overlying. This image was collected following the filamentation process, but prior to final verification process. Note the erroneous inclusion (upper-righthand portion of the vascular tree) of artifact, which can be indentified visually due to a lack of underlying native stain.

The final filament pathway was then overlaid over the original z-stack allowing for the manual removal of erroneous branches (Figure 6).

Figure 6: Maximum-intensity projection of the vasculature of the NHP placenta (CD31 - red) with the modeled vasculature overlying (grey). The modeled vasculature has been artifically set to appear skeletonized so that the underlying native-stain can be better appreciated. This verification step allows the user to confirm visually that the modeled vasculature pathways correctly stay within the bounds of the native-stained tissue. Erroneous branches have been removed at this step allowing for the calculation of the final statistics by Imaris.

2.2.3 – Data Selection

The variables of interest (Table 1 & Figure 7) were then calculated and exported into a .csv file for analysis.

Figure 7: Schematic depicting the calculation of the number of Sholl intersections. From the orgin of the vascular tree, concentric spheres are created - each with a radius of 1um greater then the previous. The number of vascular intersections with the spheres are then plotted as a function of distances from the origin.

3. Quantitative Analysis

3.1 – Preparation of Data

3.1.1 – Organization

3.1.1.1 – Vascular Segmentation Error

With generation of the proposed vascular tree, Imaris® labels each vascular segment (i.e. the vessel bound between two branching points) with a specific identifier which is consistent throughout the dataset. This means that, for any given vascular segment, the length, surface area, volume, straightness, and depth could be determined, and questions regarding the changes in vessel caliber (e.g. the percent change in the surface area per vascular segment at each depth from the origin to the distal termini) could potentially be explored.

Unfortunately, Imaris® makes two errors when identifying vascular segments which make these analyses unreliable for the purposes of this study. For the first error, the filamentation algorithm is unable to accommodate higher order vascular branching patterns (e.g. tertiary or quaternary splits), and instead erroneously inserts a small vascular segment between two closely spaced vascular branches. This error would be expected to create a population of very small vascular segments that would affect any length/surface area/volume per segment calculations as well as any use of branching depth to group vascular segments. For the second error, it was noted that Imaris® was artificially sub-dividing longer vascular segments into multiple smaller segments. This error was unpredictable in how the sub-divisions were generated, and therefore would be expected to affect all “per segment” analyses, any use of branching depth to group the vascular segments, and any straightness calculations. Given these limitations, all variables and calculations that were dependent upon vascular segment data or vascular depth were omitted.

3.1.1.2 – Organization of Remaining Data

With the exclusion of the segment-based data, the analysis was narrowed to those variables, which would not be affected by the identified issues (e.g. total vascular length/surface area/volume, number of termini, branching angles, and sholl intersections).

3.1.2 – Calculation of Variables

The segment-based data were compiled with an in-house MATLAB (MathWorks, Natick, Massachusetts, USA) program; followed by the calculation of variables of interest (Table 2). The vascular density calculations (i.e. sholl intersections) were plotted to display the number of intersections with respect to the distance from their origin (Figure 7). Additionally, the sholl intersections were cumulatively summed from their origin to their maximum radius. These data were normalized based on the total number of sholl intersection and their maximum radial distance. The rate of change of the cumulative sholl intersections with respect to the distance from the origin was computed using an in-house MATLAB algorithm implementing a five-point stencil numerical methods technique.

Table 2: Calculated Variables of Interest


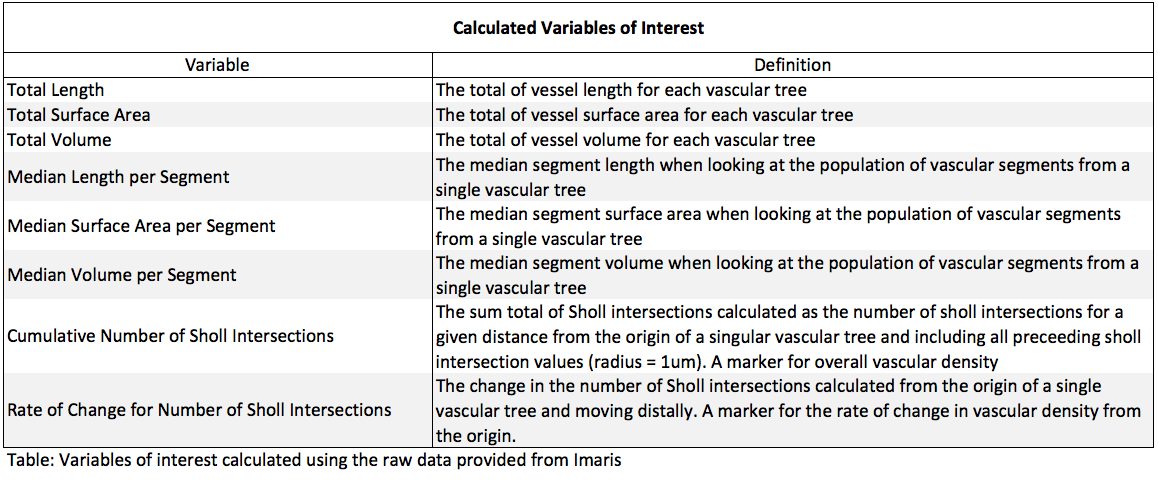


3.2 – Multivariate Linear Regression Models

Prior to generating the multivariate linear regression models, all variables were collected (Table 1), calculated (Table 2), and demographics/covariates were compiled (Table 3).

Table 3: Covariates of Interest


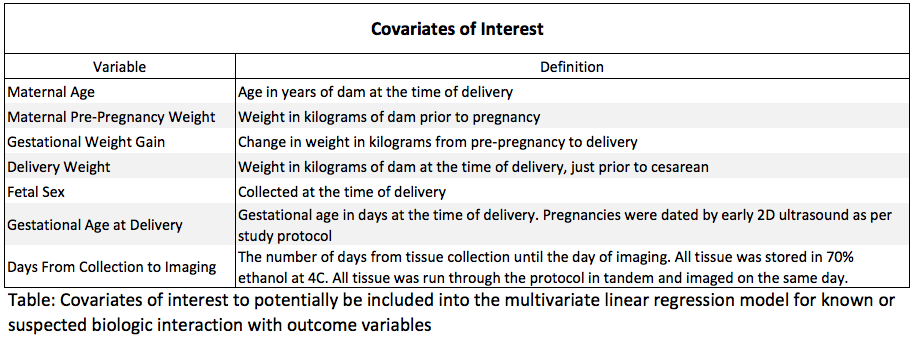


3.2.1 – Correlation Table

To appreciate the relationships between our variables of interest, scatter plots were generated and correlation coefficients generated. Significance was assessed using a threshold p-value <0.05.

3.2.2 – Univariate Regression Models

Unadjusted relationships between Group Assignment (Control Vs. Intervention) and outcomes were assessed using univariate regression modeling. Continuous outcomes were assessed with univariate linear regression models while nominal outcomes (e.g. fetal sex) were assessed using univariate logistic regression models. Those outcomes found to have a significant association with Group Assignment were controlled for in the multivariate models.

3.2.3 – Selection of Covariates for the Multivariate Linear Regression Models

Covariates of interest were selected from identified, known or suspected biologic relationship with the outcomes of interest (Table 3). Characteristics assessed included maternal age at delivery, maternal pre-pregnancy weight, maternal gestational weight gain, and maternal delivery weight, fetal sex, gestational age at delivery, and days from tissue collection to imaging.

3.2.4 – Generation of the Final Models

Multivariate linear regression models were generated including the Group-Assignment (Control Vs. Intervention) as well as the selected covariates of interest as the independent variables. Separate models were run for each of the outcomes of interest and the β, Standard Error, and 95% Confidence Intervals calculated.

3.3 – Study Strengths and Limitations

What we have presented is a novel deep-tissue imaging and vascular quantification protocol developed for use on clarified and immunofluorescently stained NHP placental tissue. Quantified analysis of placental micro-vasculature can provide a standardized and reproducible pathway to analyze and compare the large amount of complex spatial and architectural data available. While there is great potential for this approach, our protocol has many strengths and limitations that must be understood prior to utilization.

3.3.1 – Strengths and Limitations in the Development of a Novel Standardized Protocol

With the development of any novel protocol, we were limited in our ability to start with a power calculation as well as the *a priori* hypotheses we could test.

The decision was made to develop a standardized protocol for both imaging and vascular quantification. This was done to minimize subjectivity and inter-observer variability in order to allow for utilization and validation from other groups. The use of a one-size-fits-all approach for filamentation generation allows for the potential incorporation of noise into the dataset that otherwise might have been minimized through individualization of the filamentation parameters, thus increasing the potential for a Type 2 error.

As with the development of any novel protocol, validation of the methods will come with time and utilization.

3.3.2 – Strengths and Limitations of the Tissue Collection

While some investigators have found that the micro-architecture of the placenta displays regional heterogeneity, the precise differences – as they impact the variables collected through this protocol – are not known at this time. We were therefore unable to account for any potential differences in the location of selected placental biopsies that may effect the resultant output, thereby making any future comparative studies vulnerable to the unknown impact of spatial heterogeneity on the quantified results.

3.3.3 – Strength and Limitations of Image Collection

By imaging all included tissue as a single cohort, the spinning disk confocal can limit the time effect as it impacts tissue imaged *en block*. With overall image times of ~6 hours for 60 pieces of tissue, any deterioration of the immuno-fluorescent staining between the first and last pieces of tissue imaged was therefore minimized.

Unfortunately, Imaris® is not able to easily quantify the volume of space occupied by the stained tissue. While the villous trophoblast can be labeled and imaged using CK7 (as seen in Figure 1), this volume of space occupied by the stain cannot be reliably captured using either the Imaris® “Surface Generation” or “Filamentation” modules. Additionally, while both the size of the tissue biopsy and the size of the visual field can be standardized, the orientation of the tissue inside the z-stack cannot be standardized. This prevents the user from being able to crop the size of the visual field to include only the tissue of interest and no surrounding free space. Tissue that is orientated at 45 degrees from the horizontal plane would therefore incorporate a large amount of surrounding free space due to the oblique orientation when compared to a piece of tissue oriented along the horizontal plane. Without knowing the amount of space that the tissue occupies, we are therefore unable to calculate density variables that would be of potential interest (e.g. number of branches/um^3^, vascular surface area/um^3^, etc.).

Lastly, it cannot be guaranteed that each z-stack collected includes a stem villous and it’s entire vascular tree. Without being able to objectively standardize the origin and scope of each vascular tree being assessed, we are limited in our ability to directly compare some parameters (e.g. branch depth, number of termini, etc.) between tissues. We were able to attempt to correct for this by calculating variables to assess for the percent change in caliber moving from the stem villous to the distal terminus, but this approach has it’s own limitations as outlined above.

3.3.4 – Strengths and Limitations of the Imaris® Filamentation Process

The Imaris® filamentation process has its own limitations. Recognizing that this module was developed for the assessment of neuronal axons, the fundamental assumptions of the algorithm have to be understood and accounted for when applying this to vasculature.

Ideally, the “Loops” algorithm could be used for the filamentation process as this would account for the capillary beds at the distal villous termini. Unfortunately, this algorithm cannot be used for the large datasets provided with deep tissue imaging, at this time. The use of the “no loops” algorithm therefore will incorporate noise into the dataset: a single vascular loop at the villous terminus would be modeled as two separate vascular branches which approach each other to create an incomplete loop. This artifact would be anticipated to affect all imaged tissues equally.

As outlined previously, the Imaris® filamentation algorithm is able to accurately identify and trace vascular branches, however it is not able to account for a three-way (or higher order) branch. This artifact makes vascular segment and depth data unreliable, and could also bias the datasets collected if test conditions resulted in the tissue have unequal propensities for higher-order branching patters.

**Authors’ Note**

The work presented in this manuscript was performed at the Oregon National Primate Research Center (ONPRC). The ONPRC abides by the Animal Welfare Act and regulations enforced by the USDA.

**Declaration of Conflicting Interests**

The author(s) declared no potential conflicts of interest with respect to the research, authorship, and/or publication of this article. No support – aside from technical assistance – was provided to any author or related institution by either of the Visikol^®^ or Imaris^®^ Bitplane^®^ corporations.

**Funding**

The author(s) disclosed receipt of the following financial support for the research, authorship, and/or publication of this article: Funding for this project was provided by The Bill & Melinda Gates Foundation

(OPP1110865) and NIH R01 HD086331 (Frias). In addition, research reported in this publication was supported by the Office of the Director, of the National Institutes of Health under Award Number P51OD011092 to the Oregon National Primate Research Center. The content is solely the responsibility of the authors and does not necessarily represent the official views of the National Institutes of Health.

**References**

1 – Sargent JA, Roberts V, Gaffney JE, Frias AE. Clarification and confocal imaging of the nonhuman primate placental micro-anatomy. Biotechniques. 2019;66(2): 79-84.
