# Supplementary figures and images for: Micro-anatomic alterations of the placenta in a non-human primate model of gestational protein-restriction
Source: PLoS One. 2020 Jul 23;15(7):e0235840. doi: 10.1371/journal.pone.0235840 (PMC7377450; doi:10.1371/journal.pone.0235840)

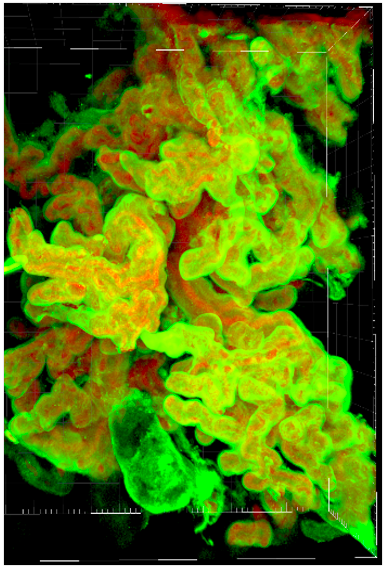

Supplement: S1 Fig — (TIFF) [file pone.0235840.s002.tiff]

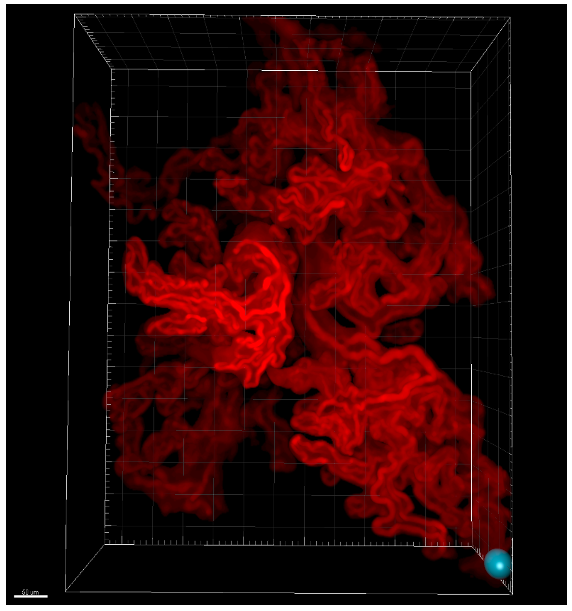

Supplement: S2 Fig — (TIFF) [file pone.0235840.s003.tiff]

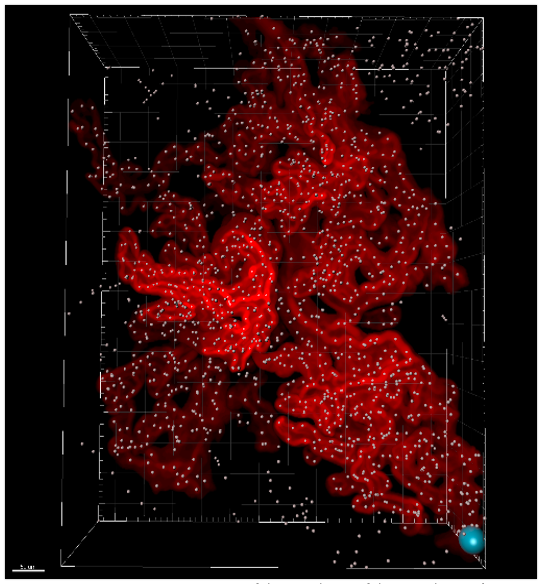

Supplement: S3 Fig — (TIFF) [file pone.0235840.s004.tiff]

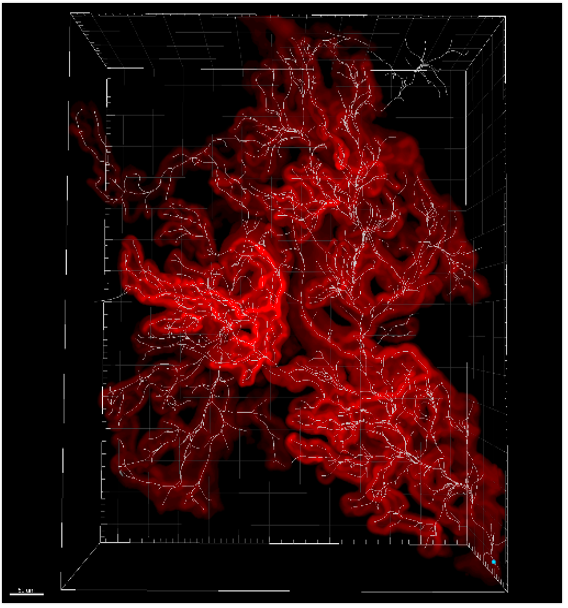

Supplement: S4 Fig — (TIFF) [file pone.0235840.s005.tiff]

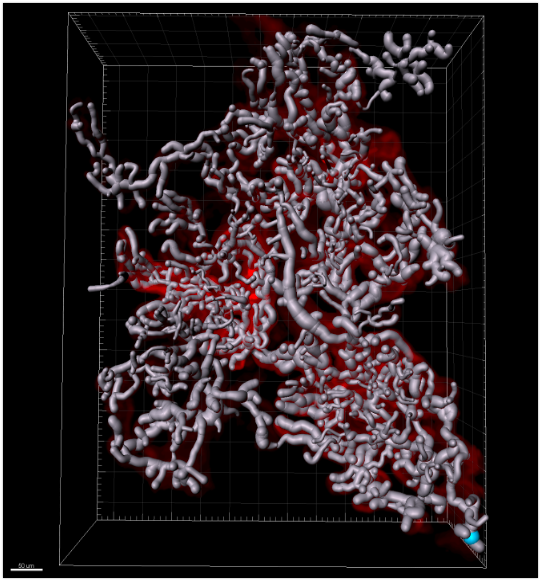

Supplement: S5 Fig — (TIFF) [file pone.0235840.s006.tiff]

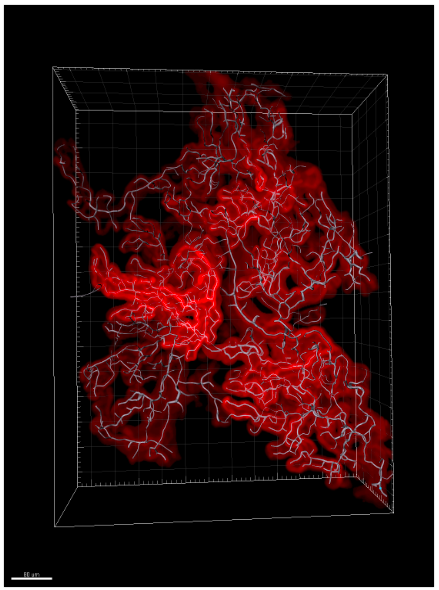

Supplement: S6 Fig — (TIFF) [file pone.0235840.s007.tiff]

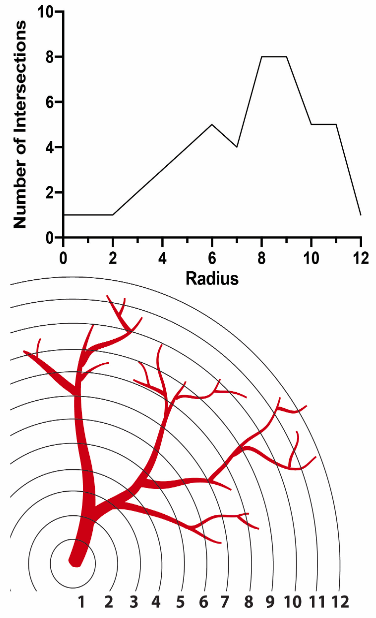

Supplement: S7 Fig — (TIFF) [file pone.0235840.s008.tiff]
